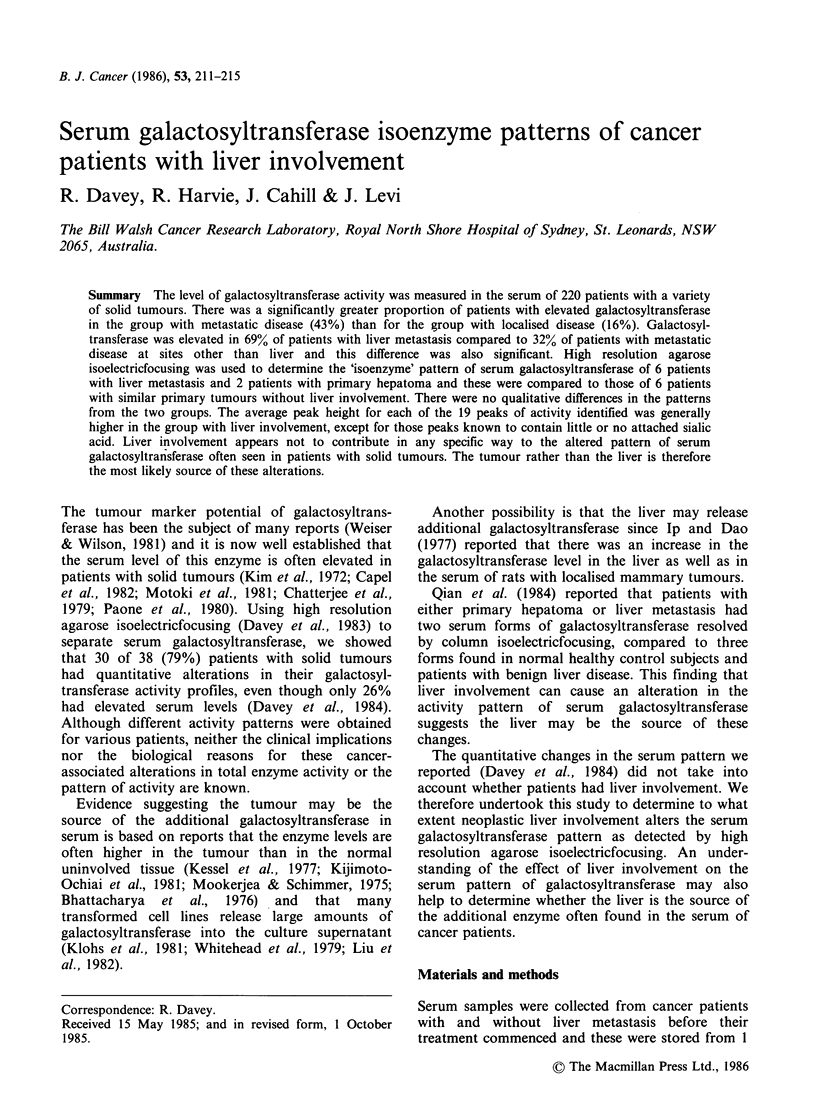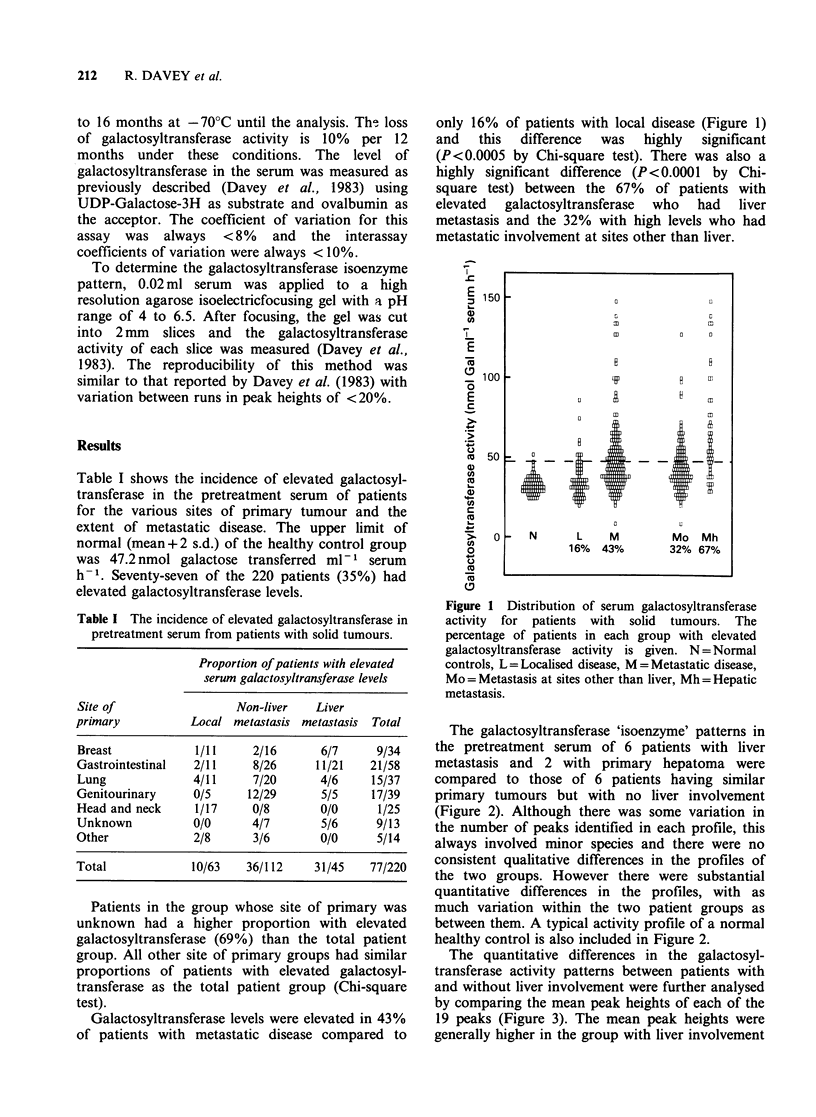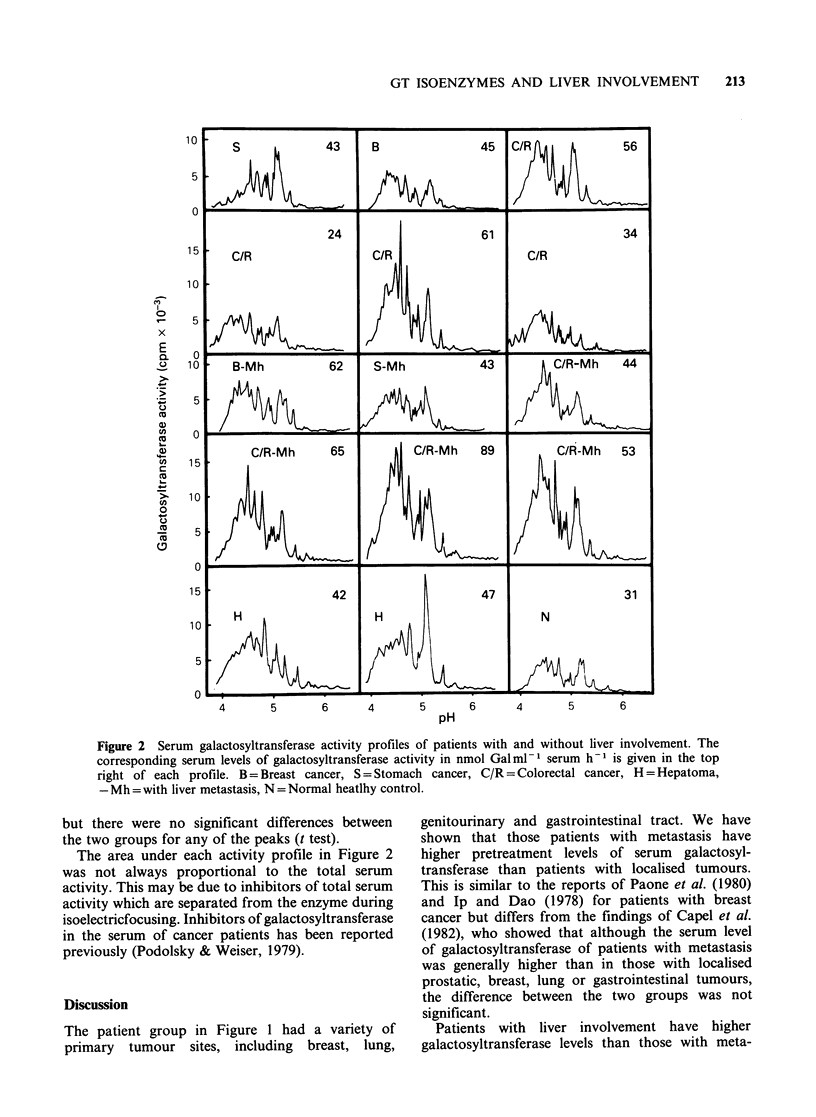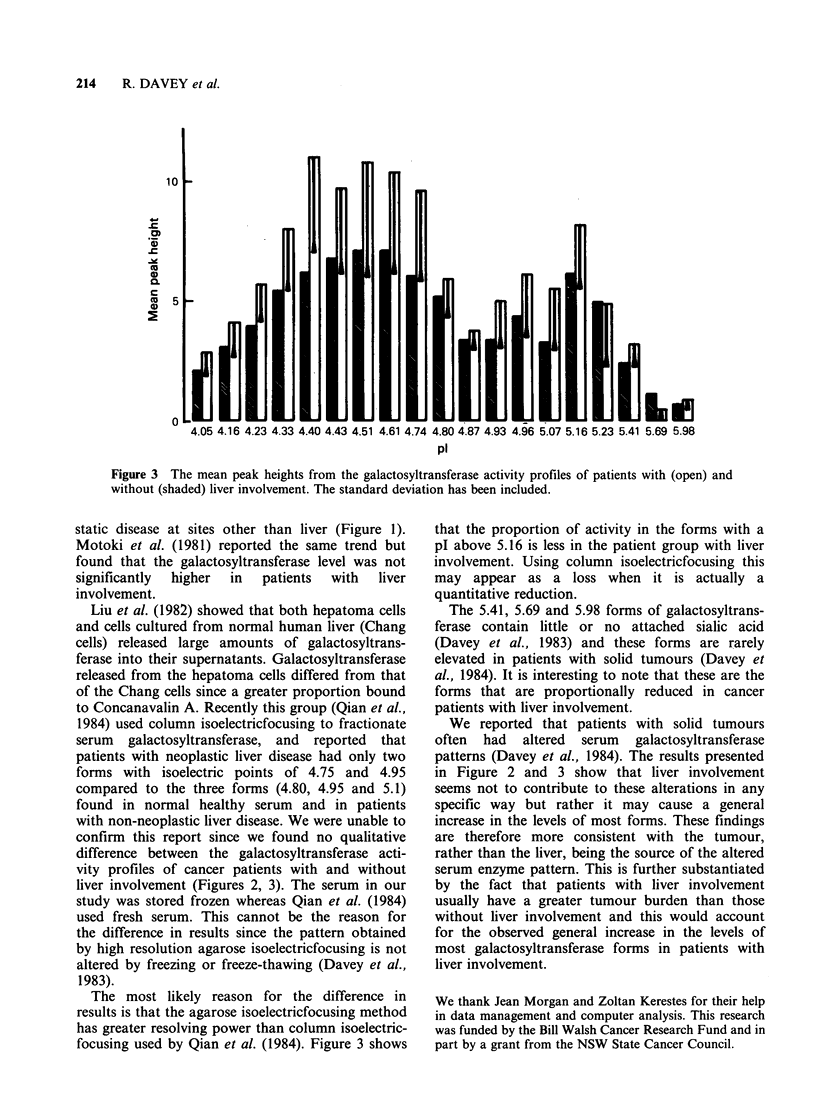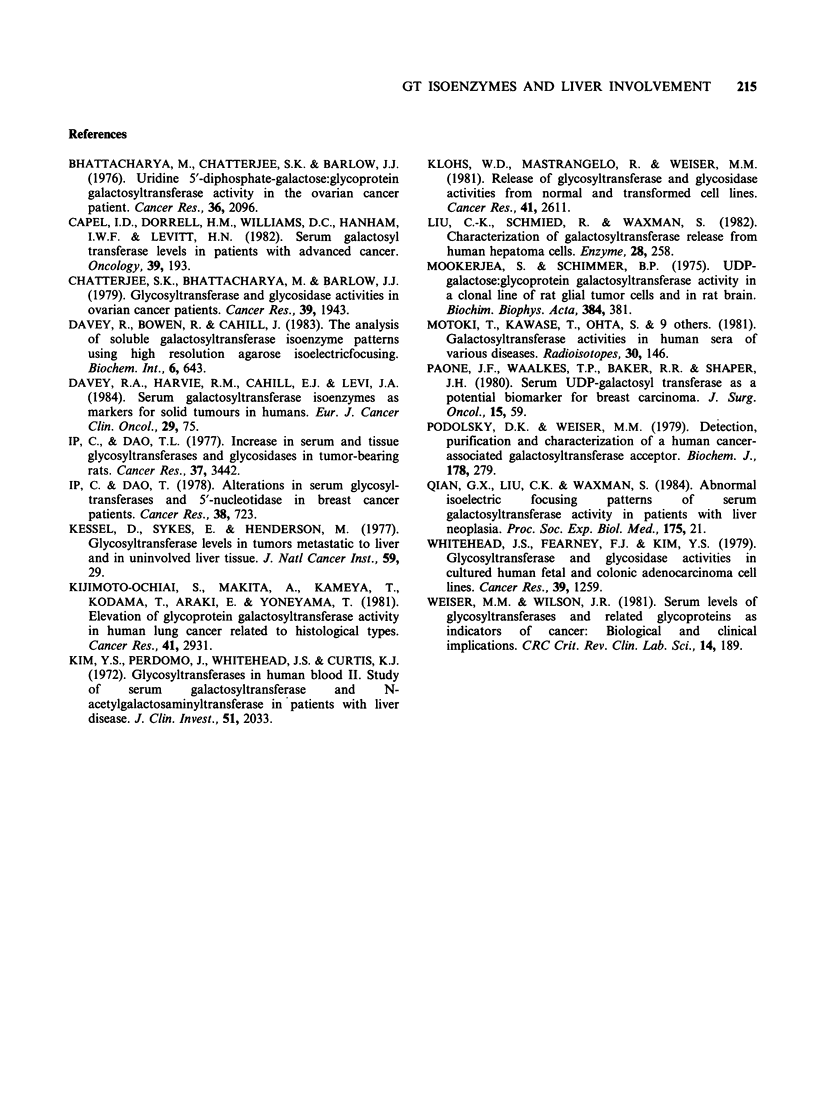# Serum galactosyltransferase isoenzyme patterns of cancer patients with liver involvement.

**DOI:** 10.1038/bjc.1986.37

**Published:** 1986-02

**Authors:** R. Davey, R. Harvie, J. Cahill, J. Levi

## Abstract

The level of galactosyltransferase activity was measured in the serum of 220 patients with a variety of solid tumours. There was a significantly greater proportion of patients with elevated galactosyltransferase in the group with metastatic disease (43%) than for the group with localised disease (16%). Galactosyltransferase was elevated in 69% of patients with liver metastasis compared to 32% of patients with metastatic disease at sites other than liver and this difference was also significant. High resolution agarose isoelectric-focusing was used to determine the 'isoenzyme' pattern of serum galactosyltransferase of 6 patients with liver metastasis and 2 patients with primary hepatoma and these were compared to those of 6 patients with similar primary tumours without liver involvement. There were no qualitative differences in the patterns from the two groups. The average peak height for each of the 19 peaks of activity identified was generally higher in the group with liver involvement, except for those peaks known to contain little or no attached sialic acid. Liver involvement appears not to contribute in any specific way to the altered pattern of serum galactosyltransferase often seen in patients with solid tumours. The tumour rather than the liver is therefore the most likely source of these alterations.


*B. J. Cancer* (1986), **53**, 211–215

### Serum galactosyltransferase isoenzyme patterns of cancer patients with liver involvement

R. Davey, R. Harvie, J. Cahill & J. Levi

*The Bill Walsh Cancer Research Laboratory, Royal North Shore Hospital of Sydney, St. Leonards, NSW 2065, Australia.*

**Summary** The level of galactosyltransferase activity was measured in the serum of 220 patients with a variety of solid tumours. There was a significantly greater proportion of patients with elevated galactosyltransferase in the group with metastatic disease (43%) than for the group with localised disease (16%). Galactosyltransferase was elevated in 69% of patients with liver metastasis compared to 32% of patients with metastatic disease at sites other than liver and this difference was also significant. High resolution agarose isoelectricfocusing was used to determine the 'isoenzyme' pattern of serum galactosyltransferase of 6 patients with liver metastasis and 2 patients with primary hepatoma and these were compared to those of 6 patients with similar primary tumours without liver involvement. There were no qualitative differences in the patterns from the two groups. The average peak height for each of the 19 peaks of activity identified was generally higher in the group with liver involvement, except for those peaks known to contain little or no attached sialic acid. Liver involvement appears not to contribute in any specific way to the altered pattern of serum galactosyltransferase often seen in patients with solid tumours. The tumour rather than the liver is therefore the most likely source of these alterations.

The tumour marker potential of galactosyltransferase has been the subject of many reports (Weiser & Wilson, 1981) and it is now well established that the serum level of this enzyme is often elevated in patients with solid tumours (Kim *et al.*, 1972; Capel *et al.*, 1982; Motoki *et al.*, 1981; Chatterjee *et al.*, 1979; Paone *et al.*, 1980). Using high resolution agarose isoelectricfocusing (Davey *et al.*, 1983) to separate serum galactosyltransferase, we showed that 30 of 38 (79%) patients with solid tumours had quantitative alterations in their galactosyltransferase activity profiles, even though only 26% had elevated serum levels (Davey *et al.*, 1984). Although different activity patterns were obtained for various patients, neither the clinical implications nor the biological reasons for these cancer-associated alterations in total enzyme activity or the pattern of activity are known.

Evidence suggesting the tumour may be the source of the additional galactosyltransferase in serum is based on reports that the enzyme levels are often higher in the tumour than in the normal uninvolved tissue (Kessel *et al.*, 1977; Kijimoto-Ochiai *et al.*, 1981; Mookerjea & Schimmer, 1975; Bhattacharya *et al.*, 1976) and that many transformed cell lines release large amounts of galactosyltransferase into the culture supernatant (Klohs *et al.*, 1981; Whitehead *et al.*, 1979; Liu *et al.*, 1982).

Correspondence: R. Davey.
Received 15 May 1985; and in revised form, 1 October 1985.

Another possibility is that the liver may release additional galactosyltransferase since Ip and Dao (1977) reported that there was an increase in the galactosyltransferase level in the liver as well as in the serum of rats with localised mammary tumours.

Qian *et al.* (1984) reported that patients with either primary hepatoma or liver metastasis had two serum forms of galactosyltransferase resolved by column isoelectricfocusing, compared to three forms found in normal healthy control subjects and patients with benign liver disease. This finding that liver involvement can cause an alteration in the activity pattern of serum galactosyltransferase suggests the liver may be the source of these changes.

The quantitative changes in the serum pattern we reported (Davey *et al.*, 1984) did not take into account whether patients had liver involvement. We therefore undertook this study to determine to what extent neoplastic liver involvement alters the serum galactosyltransferase pattern as detected by high resolution agarose isoelectricfocusing. An understanding of the effect of liver involvement on the serum pattern of galactosyltransferase may also help to determine whether the liver is the source of the additional enzyme often found in the serum of cancer patients.

### Materials and methods

Serum samples were collected from cancer patients with and without liver metastasis before their treatment commenced and these were stored from 1

© The Macmillan Press Ltd., 1986

to 16 months at −70°C until the analysis. The loss of galactosyltransferase activity is 10% per 12 months under these conditions. The level of galactosyltransferase in the serum was measured as previously described (Davey *et al.*, 1983) using UDP-Galactose-3H as substrate and ovalbumin as the acceptor. The coefficient of variation for this assay was always <8% and the interassay coefficients of variation were always <10%.

To determine the galactosyltransferase isoenzyme pattern, 0.02 ml serum was applied to a high resolution agarose isoelectricfocusing gel with a pH range of 4 to 6.5. After focusing, the gel was cut into 2 mm slices and the galactosyltransferase activity of each slice was measured (Davey *et al.*, 1983). The reproducibility of this method was similar to that reported by Davey *et al.* (1983) with variation between runs in peak heights of <20%.

### Results

Table I shows the incidence of elevated galactosyltransferase in the pretreatment serum of patients for the various sites of primary tumour and the extent of metastatic disease. The upper limit of normal (mean + 2 s.d.) of the healthy control group was 47.2 nmol galactose transferred $ml^{-1}$ serum $h^{-1}$. Seventy-seven of the 220 patients (35%) had elevated galactosyltransferase levels.

**Table I** The incidence of elevated galactosyltransferase in pretreatment serum from patients with solid tumours.

| Site of primary | *Proportion of patients with elevated serum galactosyltransferase levels* Local | Non-liver metastasis | Liver metastasis | Total |
|---|---|---|---|---|
| Breast | 1/11 | 2/16 | 6/7 | 9/34 |
| Gastrointestinal | 2/11 | 8/26 | 11/21 | 21/58 |
| Lung | 4/11 | 7/20 | 4/6 | 15/37 |
| Genitourinary | 0/5 | 12/29 | 5/5 | 17/39 |
| Head and neck | 1/17 | 0/8 | 0/0 | 1/25 |
| Unknown | 0/0 | 4/7 | 5/6 | 9/13 |
| Other | 2/8 | 3/6 | 0/0 | 5/14 |
| Total | 10/63 | 36/112 | 31/45 | 77/220 |

Patients in the group whose site of primary was unknown had a higher proportion with elevated galactosyltransferase (69%) than the total patient group. All other site of primary groups had similar proportions of patients with elevated galactosyltransferase as the total patient group (Chi-square test).

Galactosyltransferase levels were elevated in 43% of patients with metastatic disease compared to only 16% of patients with local disease (Figure 1) and this difference was highly significant ($P<0.0005$ by Chi-square test). There was also a highly significant difference ($P<0.0001$ by Chi-square test) between the 67% of patients with elevated galactosyltransferase who had liver metastasis and the 32% with high levels who had metastatic involvement at sites other than liver.

**Figure 1** Distribution of serum galactosyltransferase activity for patients with solid tumours. The percentage of patients in each group with elevated galactosyltransferase activity is given. N = Normal controls, L = Localised disease, M = Metastatic disease, Mo = Metastasis at sites other than liver, Mh = Hepatic metastasis.

The galactosyltransferase 'isoenzyme' patterns in the pretreatment serum of 6 patients with liver metastasis and 2 with primary hepatoma were compared to those of 6 patients having similar primary tumours but with no liver involvement (Figure 2). Although there was some variation in the number of peaks identified in each profile, this always involved minor species and there were no consistent qualitative differences in the profiles of the two groups. However there were substantial quantitative differences in the profiles, with as much variation within the two patient groups as between them. A typical activity profile of a normal healthy control is also included in Figure 2.

The quantitative differences in the galactosyltransferase activity patterns between patients with and without liver involvement were further analysed by comparing the mean peak heights of each of the 19 peaks (Figure 3). The mean peak heights were generally higher in the group with liver involvement

**Figure 2** Serum galactosyltransferase activity profiles of patients with and without liver involvement. The corresponding serum levels of galactosyltransferase activity in nmol Gal ml$^{-1}$ serum h$^{-1}$ is given in the top right of each profile. B = Breast cancer, S = Stomach cancer, C/R = Colorectal cancer, H = Hepatoma, – Mh = with liver metastasis, N = Normal heatlhy control.

but there were no significant differences between the two groups for any of the peaks (*t* test).

The area under each activity profile in Figure 2 was not always proportional to the total serum activity. This may be due to inhibitors of total serum activity which are separated from the enzyme during isoelectricfocusing. Inhibitors of galactosyltransferase in the serum of cancer patients has been reported previously (Podolsky & Weiser, 1979).

### Discussion

The patient group in Figure 1 had a variety of primary tumour sites, including breast, lung, genitourinary and gastrointestinal tract. We have shown that those patients with metastasis have higher pretreatment levels of serum galactosyltransferase than patients with localised tumours. This is similar to the reports of Paone *et al.* (1980) and Ip and Dao (1978) for patients with breast cancer but differs from the findings of Capel *et al.* (1982), who showed that although the serum level of galactosyltransferase of patients with metastasis was generally higher than in those with localised prostatic, breast, lung or gastrointestinal tumours, the difference between the two groups was not significant.

Patients with liver involvement have higher galactosyltransferase levels than those with meta-

**Figure 3** The mean peak heights from the galactosyltransferase activity profiles of patients with (open) and without (shaded) liver involvement. The standard deviation has been included.

static disease at sites other than liver (Figure 1). Motoki *et al.* (1981) reported the same trend but found that the galactosyltransferase level was not significantly higher in patients with liver involvement.

Liu *et al.* (1982) showed that both hepatoma cells and cells cultured from normal human liver (Chang cells) released large amounts of galactosyltransferase into their supernatants. Galactosyltransferase released from the hepatoma cells differed from that of the Chang cells since a greater proportion bound to Concanavalin A. Recently this group (Qian *et al.*, 1984) used column isoelectricfocusing to fractionate serum galactosyltransferase, and reported that patients with neoplastic liver disease had only two forms with isoelectric points of 4.75 and 4.95 compared to the three forms (4.80, 4.95 and 5.1) found in normal healthy serum and in patients with non-neoplastic liver disease. We were unable to confirm this report since we found no qualitative difference between the galactosyltransferase activity profiles of cancer patients with and without liver involvement (Figures 2, 3). The serum in our study was stored frozen whereas Qian *et al.* (1984) used fresh serum. This cannot be the reason for the difference in results since the pattern obtained by high resolution agarose isoelectricfocusing is not altered by freezing or freeze-thawing (Davey *et al.*, 1983).

The most likely reason for the difference in results is that the agarose isoelectricfocusing method has greater resolving power than column isoelectricfocusing used by Qian *et al.* (1984). Figure 3 shows that the proportion of activity in the forms with a pI above 5.16 is less in the patient group with liver involvement. Using column isoelectricfocusing this may appear as a loss when it is actually a quantitative reduction.

The 5.41, 5.69 and 5.98 forms of galactosyltransferase contain little or no attached sialic acid (Davey *et al.*, 1983) and these forms are rarely elevated in patients with solid tumours (Davey *et al.*, 1984). It is interesting to note that these are the forms that are proportionally reduced in cancer patients with liver involvement.

We reported that patients with solid tumours often had altered serum galactosyltransferase patterns (Davey *et al.*, 1984). The results presented in Figure 2 and 3 show that liver involvement seems not to contribute to these alterations in any specific way but rather it may cause a general increase in the levels of most forms. These findings are therefore more consistent with the tumour, rather than the liver, being the source of the altered serum enzyme pattern. This is further substantiated by the fact that patients with liver involvement usually have a greater tumour burden than those without liver involvement and this would account for the observed general increase in the levels of most galactosyltransferase forms in patients with liver involvement.

We thank Jean Morgan and Zoltan Kerestes for their help in data management and computer analysis. This research was funded by the Bill Walsh Cancer Research Fund and in part by a grant from the NSW State Cancer Council.

### References

BHATTACHARYA, M., CHATTERJEE, S.K. & BARLOW, J.J. (1976). Uridine 5′-diphosphate-galactose:glycoprotein galactosyltransferase activity in the ovarian cancer patient. *Cancer Res.*, **36**, 2096.

CAPEL, I.D., DORRELL, H.M., WILLIAMS, D.C., HANHAM, I.W.F. & LEVITT, H.N. (1982). Serum galactosyl transferase levels in patients with advanced cancer. *Oncology*, **39**, 193.

CHATTERJEE, S.K., BHATTACHARYA, M. & BARLOW, J.J. (1979). Glycosyltransferase and glycosidase activities in ovarian cancer patients. *Cancer Res.*, **39**, 1943.

DAVEY, R., BOWEN, R. & CAHILL, J. (1983). The analysis of soluble galactosyltransferase isoenzyme patterns using high resolution agarose isoelectricfocusing. *Biochem. Int.*, **6**, 643.

DAVEY, R.A., HARVIE, R.M., CAHILL, E.J. & LEVI, J.A. (1984). Serum galactosyltransferase isoenzymes as markers for solid tumours in humans. *Eur. J. Cancer Clin. Oncol.*, **29**, 75.

IP, C., & DAO, T.L. (1977). Increase in serum and tissue glycosyltransferases and glycosidases in tumor-bearing rats. *Cancer Res.*, **37**, 3442.

IP, C. & DAO, T. (1978). Alterations in serum glycosyl-transferases and 5′-nucleotidase in breast cancer patients. *Cancer Res.*, **38**, 723.

KESSEL, D., SYKES, E. & HENDERSON, M. (1977). Glycosyltransferase levels in tumors metastatic to liver and in uninvolved liver tissue. *J. Natl Cancer Inst.*, **59**, 29.

KIJIMOTO-OCHIAI, S., MAKITA, A., KAMEYA, T., KODAMA, T., ARAKI, E. & YONEYAMA, T. (1981). Elevation of glycoprotein galactosyltransferase activity in human lung cancer related to histological types. *Cancer Res.*, **41**, 2931.

KIM, Y.S., PERDOMO, J., WHITEHEAD, J.S. & CURTIS, K.J. (1972). Glycosyltransferases in human blood II. Study of serum galactosyltransferase and N-acetylgalactosaminyltransferase in patients with liver disease. *J. Clin. Invest.*, **51**, 2033.

KLOHS, W.D., MASTRANGELO, R. & WEISER, M.M. (1981). Release of glycosyltransferase and glycosidase activities from normal and transformed cell lines. *Cancer Res.*, **41**, 2611.

LIU, C.-K., SCHMIED, R. & WAXMAN, S. (1982). Characterization of galactosyltransferase release from human hepatoma cells. *Enzyme*, **28**, 258.

MOOKERJEA, S. & SCHIMMER, B.P. (1975). UDP-galactose:glycoprotein galactosyltransferase activity in a clonal line of rat glial tumor cells and in rat brain. *Biochim. Biophys. Acta*, **384**, 381.

MOTOKI, T., KAWASE, T., OHTA, S. & 9 others. (1981). Galactosyltransferase activities in human sera of various diseases. *Radioisotopes*, **30**, 146.

PAONE, J.F., WAALKES, T.P., BAKER, R.R. & SHAPER, J.H. (1980). Serum UDP-galactosyl transferase as a potential biomarker for breast carcinoma. *J. Surg. Oncol.*, **15**, 59.

PODOLSKY, D.K. & WEISER, M.M. (1979). Detection, purification and characterization of a human cancer-associated galactosyltransferase acceptor. *Biochem. J.*, **178**, 279.

QIAN, G.X., LIU, C.K. & WAXMAN, S. (1984). Abnormal isoelectric focusing patterns of serum galactosyltransferase activity in patients with liver neoplasia. *Proc. Soc. Exp. Biol. Med.*, **175**, 21.

WHITEHEAD, J.S., FEARNEY, F.J. & KIM, Y.S. (1979). Glycosyltransferase and glycosidase activities in cultured human fetal and colonic adenocarcinoma cell lines. *Cancer Res.*, **39**, 1259.

WEISER, M.M. & WILSON, J.R. (1981). Serum levels of glycosyltransferases and related glycoproteins as indicators of cancer: Biological and clinical implications. *CRC Crit. Rev. Clin. Lab. Sci.*, **14**, 189.